# Fine-scale species distribution modelling and genotyping by sequencing to examine hybridisation between two narrow endemic plant species

**DOI:** 10.1038/s41598-020-58525-2

**Published:** 2020-01-31

**Authors:** J. W. Ball, T. P. Robinson, G. W. Wardell-Johnson, J. Bovill, M. Byrne, P. G. Nevill

**Affiliations:** 1 School of Earth and Planetary Sciences, Curtin University, GPO Box U1987, Perth, WA 6845 Australia; 2 ARC Centre for Mine Site Restoration and School of Molecular and Life Sciences, Curtin University, GPO Box U1987, Perth, WA 6845 Australia; 3 Centre for Australian National Biodiversity Research, National Research Collections Australia, GPO Box 1600, Canberra, ACT 2601 Australia; 4 Biodiversity and Conservation Science, Department of Biodiversity, Conservation and Attractions, Locked Bag 104, Bentley Delivery Centre, Perth, WA 6983 Australia; 5 School of Molecular and Life Sciences, Curtin University, GPO Box U1987, Perth, WA 6845 Australia

**Keywords:** Conservation biology, Environmental impact

## Abstract

Hybridization has an important and often positive role in plant evolution. However, it can also have negative consequences for species. Two closely related species of *Ornduffia* are endemic to the Porongurup Range in the South West Australian Global Biodiversity Hotspot. The rare *Ornduffia calthifolia* is found exclusively on the summits, while *O. marchantii* is more widely dispersed across a greater range of elevation and is not considered threatened. Hybridisation in suitable overlapping habitat has been suspected between them for decades. Here we combine genotyping by sequencing to verify hybridisation genetically, and fine scale (2 m resolution) species distribution modelling (SDM) to test if hybrids occur in suitable intersecting habitat. From a study area of *c*. 4700 ha, SDM identified *c*. 275 ha and *c*. 322 ha of suitable habitat for *O. calthifolia* and *O. marchantii*, respectively. We identified range overlap between species of *c*. 59 ha), which enveloped 32 individuals confirmed to be hybrids. While the hybrids were at the margin of suitable habitat for *O. marchantii*, their preference for elevated habitat was closer to the more narrowly distributed *O. calthifolia*. The combination of genetic data and fine scale spatial modelling approaches enabled a better understanding of hybridisation among taxa of conservation significance. However, the level to which hybrid proliferation and competition for habitat presents as a threat to *O. calthifolia* is currently unknown and requires priority in conservation management given the threats from global warming and disturbance by tourism.

## Introduction

Hybridisation of two genetically distinguishable populations can have constructive or destructive outcomes for taxa^1^. For example, genetic and demographic swamping are two potential evolutionary processes that may cause extinction of one or both parents. Genetic swamping is the more frequent outcome, which occurs when hybrids replace one or both parents^2,3^. Demographic swamping results from outbreeding depression; where parent taxa expend reproductive energy on infertile or unfit hybrids, leading to population growth rates below what is needed for replacement^4^. Alternatively, hybridisation may increase diversity in previously isolated, inbred, populations (genetic rescue), increasing their viability^5^.

Narrow range endemic taxa are more vulnerable to extinction from the outcomes of hybridisation than common species^4,6^. Global biodiversity hotspots contain exceptional concentrations of endemism, including almost half of all global plant biodiversity, yet cover a fraction of the Earth’s surface^7^. These hotspots are therefore priorities for conservation management. For example, high levels of plant diversity and endemism occur in the South West Australian Global Biodiversity Hotspot, which includes over 2500 threatened vascular species^8^. This flora includes many groups of closely related taxa with narrow distributions that are geographically proximal in the relatively subdued landscapes of south-western Australia. For these taxa, understanding interactions through hybridisation have until recently been limited by available technology.

Recent advances in molecular biology (e.g. next generation sequencing) and fine scale species distribution modelling (SDM) means they can now be used together to confirm hybridisation and determine suitable habitat at unprecedented levels of detail^9,10^. Genotyping by sequencing can produce thousands of high-quality markers, enabling comprehensive genetic analyses even of non-model organisms^11,12^. Opportunities for hybridisation can be readily explored using SDM, by extrapolating the environmental correlates of the parent populations^13^ and delineating their overlapping niches. While there are multiple examples of SDM complementing genomic analyses (e.g.^14,15^), few have modelled endemic species at finer than 10 m resolution^16,17^.

Two closely related species of *Ornduffia* are endemic to a single mountain range in south-western Australia. *Ornduffia calthifolia* is declared rare flora under the Commonwealth *Environment Protection and Biodiversity Conservation Act 1999*^18^ and is found exclusively on the summits of the Porongurup Range^19^. *Ornduffia marchantii* is found at lower elevations but is not considered threatened. Hybridisation potential has been confirmed in greenhouse experiments^20^ and is thought to be occurring in the intervening zone^21^. Keppel *et al*. (^22^) suggested that asymmetric hybridisation, with *O. calthifolia* as the maternal parent, could threaten the survival of *O. calthifolia*.

Here, we integrate genotyping by sequencing data and fine-scale SDM to confirm hybridisation between the two *Ornduffia* species in south-western Australia. We have three specific aims and associated hypotheses:

1. verify the occurrence of hybrid individuals in the wild;
2. estimate the pattern of hybridisation across species and populations; and
3. examine whether hybridisation occurs in suitable overlapping habitat identified by distribution modelling. We hypothesised that the distribution of hybrid individuals would be spatially variable and occur more frequently in suitable overlapping habitat

## Materials and Methods

### Study area

The Porongurup Range (670 m a.s.l.), along with the Stirling Ranges (1090 m a.s.l.), provide the only significant altitudinal relief within south-western Australia^23^. The Porongurup Range (central point: 34°40′S, 117°52′E) is Australia’s largest granite outcrop^22^ consisting of multiple granite domes about 1100 million years old^24^. It varies in elevation from *c*. 230–670 m over 26 km^2^ and has a Mediterranean climate, with cool, wet winters and hot, dry summers. Soils at the base of this range may remain moist all year. Higher elevations receive greater orographic rainfall than the base^25^, though no official measurements have been produced^26^.

### Study species

**Figure 1:**
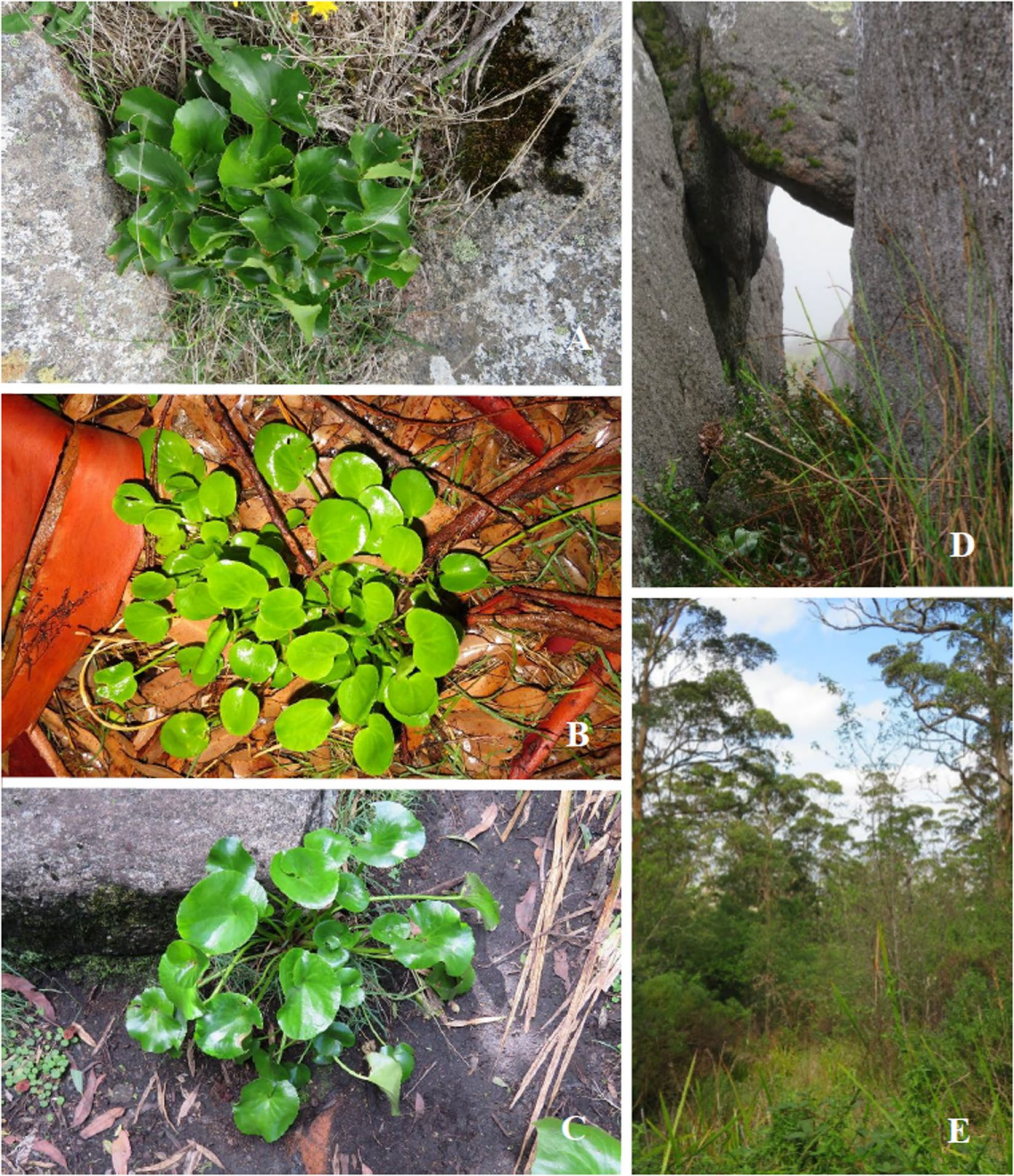
*Ornduffia* species and habitat within the Porongurup Range (non-flowering season). (**A**) *O. calthifolia*, with large fleshy leaves and distinct crenulations, found in shallow soils amongst bare rock. (**B**) *O. marchantii*, with small, smooth heart shaped leaves, found in leaf litter of forest floor. (**C**) Putative hybrid with intermediate morphological characteristics of both parent species, along the Devils Slide walking track. (**D**) Typical habitat of *O. calthifolia*, in protected crevasses between rock formations at high elevations (>550 m). (**E**) Typical habitat of *O. marchantii*, in karri (*Eucalyptus diversicolor*) forest, the dominate vegetation type at lower elevation of the Porongurup Range.

The herbaceous genus *Ornduffia* (Family: Menyanthaceae) includes eight semi-aquatic and wetland species with fleshy leaves, native to southern Australia^22^. *Ornduffia calthifolia* (Fig. 1A) is around 75 cm tall when in flower and has considerably larger leaves and habit, relative to *Ornduffia marchantii* (Fig. 1B)., which has smooth, heart-shaped leaves (Fig. 1B). *Ornduffia marchantii* is strongly distylous, making individuals self-incompatible^20^. This differs from *O. calthifolia*, which is self-compatible^27^, allowing it to produce copious fertile seed without reliance on pollinating insects^20^. Both species produce small (<2 mm) elliptic seeds that demonstrate hydrophobic properties, assisting dispersal by water^28^. Additionally, ants are thought to be an important dispersal agent for both species^28^.

**Figure 2:**
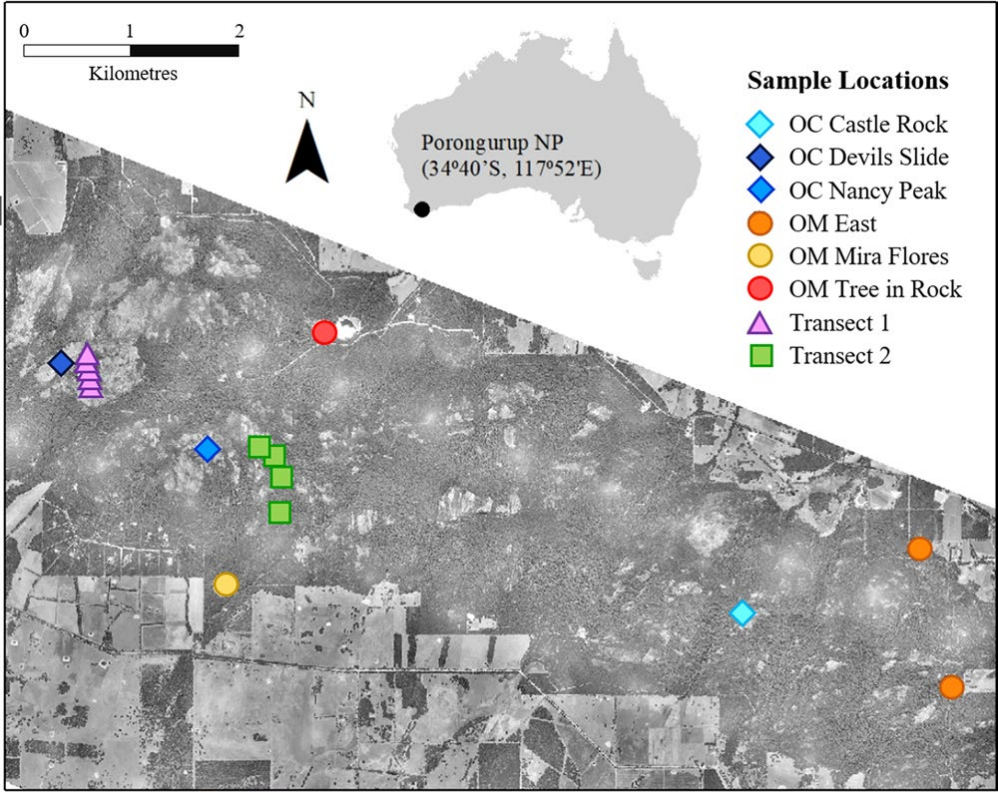
Sampling locations for *O. calthifolia*, *O. marchantii* and transect populations of putative hybrids across the Porongurup Range National Park. OC = parent populations of *O. calthifolia*, OM = parent populations of *O. marchantii*. Imagery was taken in 2011 by airplane flying 1700–2200 m above the ground using a Digital Mapping Camera from Z/I Imaging and acquiring RGB spectral bands covering 425–515, 515–550 and 600–650 nm at a spatial resolution of 0.2 m. ArcGIS v 10.5 was used to create the map.

Putative hybrids (Fig. 1C) have only been identified along a long-established walking track at Devil’s Slide (Fig. 2)^21,22^. The two parent species have different habitat preferences; *O. calthifolia* occurs mostly within moist, sheltered crevices at the highest-elevation (e.g. 640 m asl) granite peaks (Fig. 1D^18,20^), whilst *O. marchantii* occurs at lower elevations (e.g. 350 m asl), often in disturbed habitat, under forest dominated by karri (*Eucalyptus diversicolor*) where soils are wet and loamy^25,29^(Fig. 1E^20^).

### Sample collection

We conducted a field survey in the Porongurup Range in March 2017. Three reference populations for each of *O. calthifolia* (‘OC Nancy Peak’, ‘OC Devils Slide’, ‘OC Castle Rock’) and *O. marchantii* (‘OM Tree in Rock’, ‘OM Mira Flores’, ‘OM East’) were sampled (Fig. 2), and reference populations were located across the species ranges. We performed transect sampling for putative hybrids, starting at low elevations, scaling the mountains, and collecting populations when found. Transect one was sampled adjacent to a recreational walking track to Devil’s Slide and Transect two in undisturbed habitat leading to the summit of Nancy Peak (Fig. 2).

Transect one comprised five populations (T1, 1–5) of putative hybrids (Fig. 2) between 580–630 m asl. Transect one sampling was limited to within 2 m of the walking track. We ran Transect two on the south side of Nancy Peak (Fig. 2) in forest seldom traversed. This transect comprised three populations between 360 and 560 m (T2, 1–3), with two additional individuals at 600 m comprising T2, 4.

We collected 187 samples, each population comprising 13 or 14 samples except for T2,4, which only had two individuals. The largest healthy leaf from each sample was collected for morphometric analysis, placed in an airtight bag and refrigerated in the field. Where there were ample individuals, samples were a minimum of 5 m apart. We recorded the coordinates of each sample with the aid of a GNSS receiver (Garmin Etrex 10).

### Morphometric analysis

Morphometric analysis was used to identify phenological intermediates. *Ornduffia marchantii* is identified as having a smaller narrower leaf and shorter stem than *O. calthifolia*^20^. Three leaf dimensions (length along midrib from base to tip, maximum width perpendicular to midrib and maximum length from tip to lower lobe), and petiole length were measured on the largest healthy leaf of each plant. Sampling occurred out of flowering season and thus no reproductive variables could be obtained. We produced 2-dimensional multidimensional scaling (MDS) ordination plots in PATN V2.3^29^ to visually assess trends in the resultant patterns. The goal of MDS is to faithfully represent the distance matrix in the lowest possible dimensional space, with level of stress measuring the resultant distortion. If stress is low, the chosen dimensional representation (in this case two) reasonably represents the objects relative positions. We used the Gower association measure^30^ to derive the distance matrix, due to its capacity to handle continuous data^29^, with 1000 random starts, a maximum 1000 iterations and a cut value of 0.938.

We ran analysis of similarities (ANOSIM) and permutational multivariate analysis of variance (PERMANOVA) to test for significant morphological differences between these *a priori* groups in PAST3 software^31^. These functions operate directly on the dissimilarity matrix and are allied with MDS ordination in that it uses the rank order of dissimilarity values. If two groups of sampling units are different, then compositional dissimilarities between the groups ought to be greater than those within groups. The ANOSIM statistic R is based on the difference of mean ranks between groups (r_B) and within groups. In both cases the Gower dissimilarity measure and 10 000 permutations were applied.

### Genetic analysis

Diversity Arrays Technology Pty. Ltd extracted DNA from 0.1 g dried leaf samples with the NucleoSpin Plant II method (Macherey-Nagel). Genome wide scans by Diversity Arrays Technology (DArTSeq) sequenced the isolated DNA fragments. DARTSeq is a genome complexity reduction method combined with next generation sequencing technologies, capable of identifying tens of thousands of single nucleotide polymorphism (SNP) loci. To ensure only high quality and informative markers were included, loci with more than 10% missing data (call rate <0.9) were removed from analysis^32^ as were loci with a minor allele frequency <0.05 (e.g.^33^) or average read depth <5 (e.g.^34^). Loci displaying departure from Hardy-Weinberg equilibrium and linkage equilibrium were removed with the dartR package in R^35^.

We used BAYESCAN V2.1^36^ to identify outlier loci, whose allele frequencies vary more between populations than expected under genetic drift. These loci may represent putatively non-neutral genetic regions and were removed from the analysis. We applied default parameters for iterations (20 pilot runs of 5000 iterations each, 100 000 total iterations with 50 000 burn-in). We set ‘Prior odds for neutral model’ to 200, as higher odds are required for larger datasets^37^. The inbreeding coefficient was set as ‘F_IS_ uniform between 0 and 0.5’ (default is uniform between 0 and 1) based on F_IS_ results calculated for each locus using the R package ADEGENET^38^. We applied a false discovery rate (FDR) threshold of 0.05 (q value < 0.05) to separate neutral and adaptive loci^36^.

Expected heterozygosity (H_E_), observed heterozygosity (H_O_) and allelic richness (AR) values were produced in the R package HIERFSTAT^39^. Inbreeding coefficients (F_IS_) were calculated for each locus using R packages ADEGENET^38^_._ We grouped all samples into four populations for calculating genetic diversity; the two parent reference populations, Transect 1 and Transect 2.

We generated a pairwise Nei’s genetic distance^40^ matrix in the R package STAMPP^41^. From the distance matrix, we generated a PCoA ordination graph in GENALEX 6.5^42^ to visualise population structure and similarity between samples^43^. We also used Bayesian clustering methods in STRUCTURE V2.3.4^44,45^ to produce definitive identification of hybrid individuals. STRUCTURE implements a Markov chain Monte Carlo (MCMC) algorithm, calculating percentage admixture individuals to K genetic clusters. We tested for optimal number of clusters with 10 runs each of K = 1–6. We ran a burn-in of 50 000 iterations and 250 000 MCMC iterations, no previous population information and correlated allele frequencies (e.g.^46^). A subsequent analysis in STRUCTURE was performed with USEPOPINFO = 1 to allocate putative hybrid individuals to the parental populations. We used the ΔK method^47^ in STRUCTURE HARVESTER V0.6.93^48^ to delineate optimal K. Individual replicates were aligned using the ‘full search’ algorithm in CLUMPP 1.1.2^49^. The optimal K value was then used in the delineation of hybrid individuals. Pure species were delineated by a threshold at 0.1, meaning individuals assigned a value > 0.90 to a species were categorised as pure (e.g.^46^).

To identify hybrid status of individuals as back-crossed or F_1_ hybrids, we ran analyses on NEW-HYBRIDS^50^ to assign individuals to one of six genealogical classes (pure OC, pure OM, F_1_ hybrid, F_2_ hybrid, back-crossed OC and back-crossed OM) (e.g.^46^). We validated the accuracy at which both STRUCTURE and NEW-HYBRIDS correctly classified individuals using simulated populations created in HYBRIDLAB^51^. From each parent population we created 200 individuals, of which we simulated the creation of the other four genealogical classes identified by NEW-HYBRIDS. We then ran simulated samples through STRUCTURE and NEW HYBRIDS with the same parameters as the real data.

### Spatial patterns of hybridisation

We developed SDMs with MAXENT V3.3.3^13^ for both parent species to predict opportunities for hybridisation in the overlapping suitable habitat. All ‘pure’ individuals (89 *O. marchantii* and 59 *O. calthifolia* samples), as delineated by STRUCTURE, were included as presence data. We supplemented this survey with 207 *O. calthifolia* and 55 *O. marchantii* samples from surveys conducted in February 2012 and November 2013. As no coordinates were duplicated over surveys, we assumed all samples were unique. To quantify model accuracy, we calculated mean area under the curve (AUC) of receiver operating characteristic (ROC) curves^52^ with 100 bootstrap iterations. Each iteration randomly selected 20% of the points for an independent validation dataset. We did not develop an SDM for the hybrid because its current limited geographic extent comprises of only a few clustered samples and is therefore likely to result in under prediction.

Light detection and ranging (LiDAR) data for the Porongurup Range was captured in April 2011 with an airborne Leica ALS 50-II scanner. A flight height between 1700 and 2220 m resulted in *c*. 0.63 last return heights per m^2^, which were interpolated into a 2 m resolution digital elevation model (DEM). Horizontal and vertical accuracies are <0.35 m and <0.15 m respectively. Elevation, slope (first derivative of elevation), aspect (degrees from north), curvature (second derivative of elevation), solar radiation (WH/m^2^) at monthly intervals and the topographic wetness index (TWI) defined by Gessler *et al*.^53^ were derived from the DEM using the Spatial Analyst Toolbox of ArcGIS v 10.5^54^.

We calculated topographic roughness index (TRI), vertical distance to channel network (VDCN), topographic position index (TPI) and the SAGA Wetness Index (SWI) in SAGA v 2.1.4^55^. TRI^56^ is a measure of terrain heterogeneity, VDCN is a measure of local ridge heights. TPI^57^ identifies elevation relative to its neighbourhood. The SWI was used in addition to TWI because they were not significantly correlated (r = 0.4) and the SWI has predicted potential of soil moisture for cells with small vertical distance to a channel in valley floors more realistically than TWI^58^. As we recognised that *O. calthifolia* preferentially inhabits locations at or near the base of cliff faces we used a high pass filter to identify cliff edges and modelled for proximity. As *O. marchantii* is associated with the shade provided by karri over storey, we calculated a canopy height model (CHM).

There is no accepted method for selecting model thresholds other than they should be data driven^59^. We defined a threshold to classify suitable and unsuitable habitat by minimising misclassification of pseudo-absences created nearby traversed locations^60^.

## Results

### Morphometric analysis

**Figure 3:**
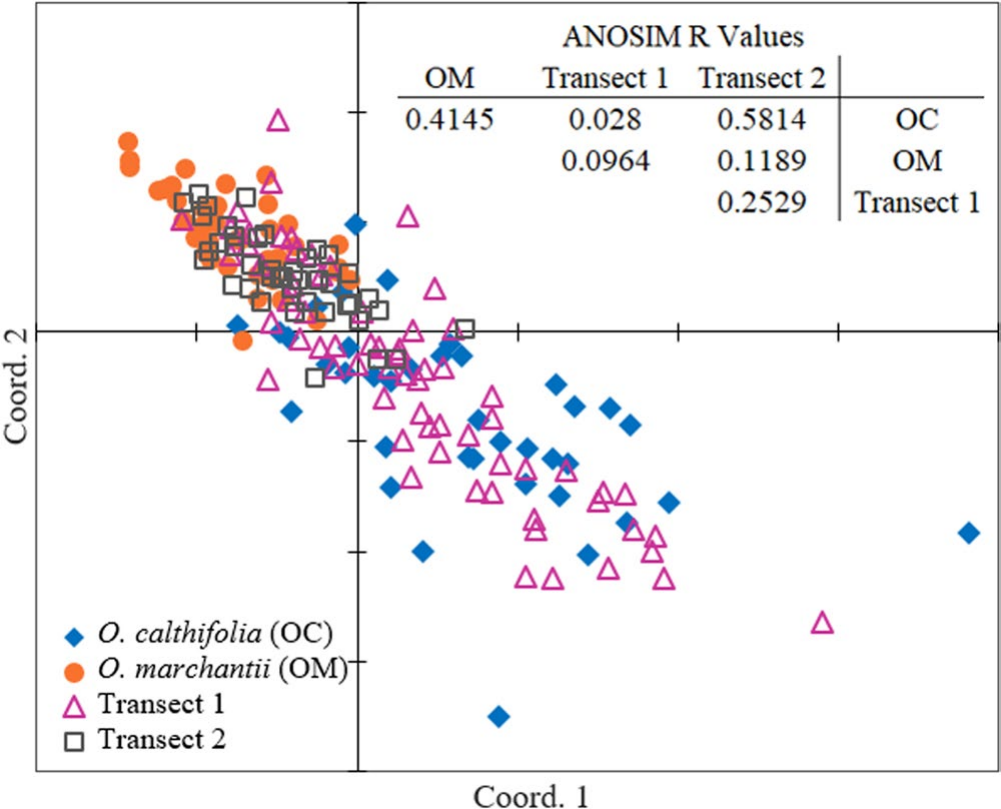
Two-dimensional ordination (Semi-strong hybrid multidimensional scaling SSH MDS, Stress = 0.129) of 187 individuals were classified into four a priori groups of *O. calthifolia*, *O. marchantii* and the two transects. R values from ANOSIM are displayed with *O. calthifolia* and Transect 1 the only populations not significantly dissimilar.

As expected, analysis of morphometric data using MDS showed separation of individuals from the two parent species classes with little overlap in ordination plot space (Fig. 3). These two species have long been recognised as separate based on morphology and habitat^20^. Samples from Transect 1 occupied broad ordination space with most individuals occurring in similar ordination space to that of *O. calthifolia*, yet also occurring with *O. marchantii* and in the space between the two species. Individuals from Transect 2 occupied ordination space between that of the two species, with some overlap with *O. marchantii*. Each class was significantly different (ANOSIM and PERMANOVA, P < 0.01) from every other class except for *O. calthifolia* with Transect 1. ANOSIM produced an overall R value of 0.2045 and PERMANOVA an F statistic of 39.76. Pairwise R values can be found in Fig. 3.

### Genetic analyses

DArT sequencing produced 48 541 SNP loci (not including presence/absence data), with 8 489 loci retained after data cleaning. BAYESCAN identified 263 outlier loci that were removed from the analysis. The more common and dispersed taxon, *O. marchantii*, had greater genetic diversity than *O. calthifolia* (H_E_ = 0.235 v. 0.083; Table 1). Further, *O. calthifolia* demonstrated departure from random mating with a much higher inbreeding coefficient than *O. marchantii* (0.651 v 0.268; Table 1). Genetic diversity estimates for samples from Transect 1 exhibited highest genetic diversity and allelic richness (Table 1). Genetic diversity estimates for Transect 2 were consistent with those for *O. marchantii*, except for a lower inbreeding coefficient (Table 1).

**Table 1:** Genetic diversity statistics for each ‘taxon’ (*Ornduffia calthifolia, O. marchantii* and plants from each of two transects), mean values across all alleles with standard deviation in brackets in the Porongurup Range, south-western Australia.

Population	N	H_E_ (δ^2^)	H_O_ (δ^2^)	AR (δ^2^)	F_IS_ (δ^2^)
*Ornduffia calthifolia*	39	0.083 (0.158)	0.033 (0.092)	1.047 (0.128)	0.651 (0.426)
*Ornduffia marchantii*	39	0.235 (0.178)	0.179 (0.151)	1.21 (0.12)	0.268 (0.375)
Transect 1	67	0.303 (0.191)	0.206 (0.133)	1.556 (0.389)	0.286 (0.289)
Transect 2	42	0.221 (0.178)	0.182 (0.16)	1.243 (0.229)	0.153 (0.359)

Number of samples (N), expected heterozygosity (H_E_), observed heterozygosity (H_O_), Allelic richness (AR) and inbreeding coefficient (F_IS_), variance (δ^2^).

**Figure 4:**
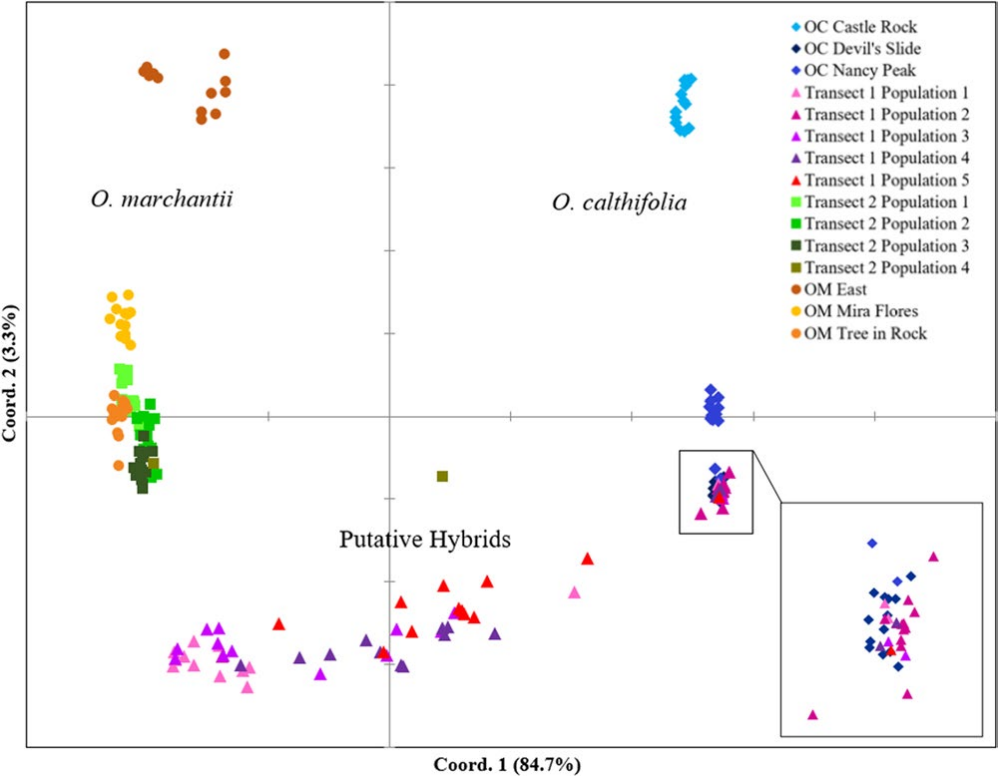
(**A**) Two-dimensional ordination (Principal coordinates analysis - PCoA) of 187 individuals from 15 populations of *Ornduffia calthifolia*, *O. marchantii* and two transects in the Porongurup Range, south-western Australia based on genetic distance. (**B**) Enlargement of inset.

There was greater differentiation between populations based on genetic distance analysis than morphometric analysis (Fig. 4). The PCoA showed *O. calthifolia* populations clustering separately from those of *O. marchantii* on the axis for coordinate 1. There was some variation within both species that was predominantly identified by coordinate 2, with the more isolated eastern populations (‘OC Castle Rock’ and ‘OM East’) exhibiting greatest separation from other populations within each species. Thirty-six individuals from Transect 1 were clustered with *O. calthifolia* from the Devil’s slide population but the majority occurred in intermediate space between the two species (Fig. 4). All samples from Transect 2 clustered with samples from *O. marchantii*, except for one that was between the two species (Fig. 4).

**Figure 5:**
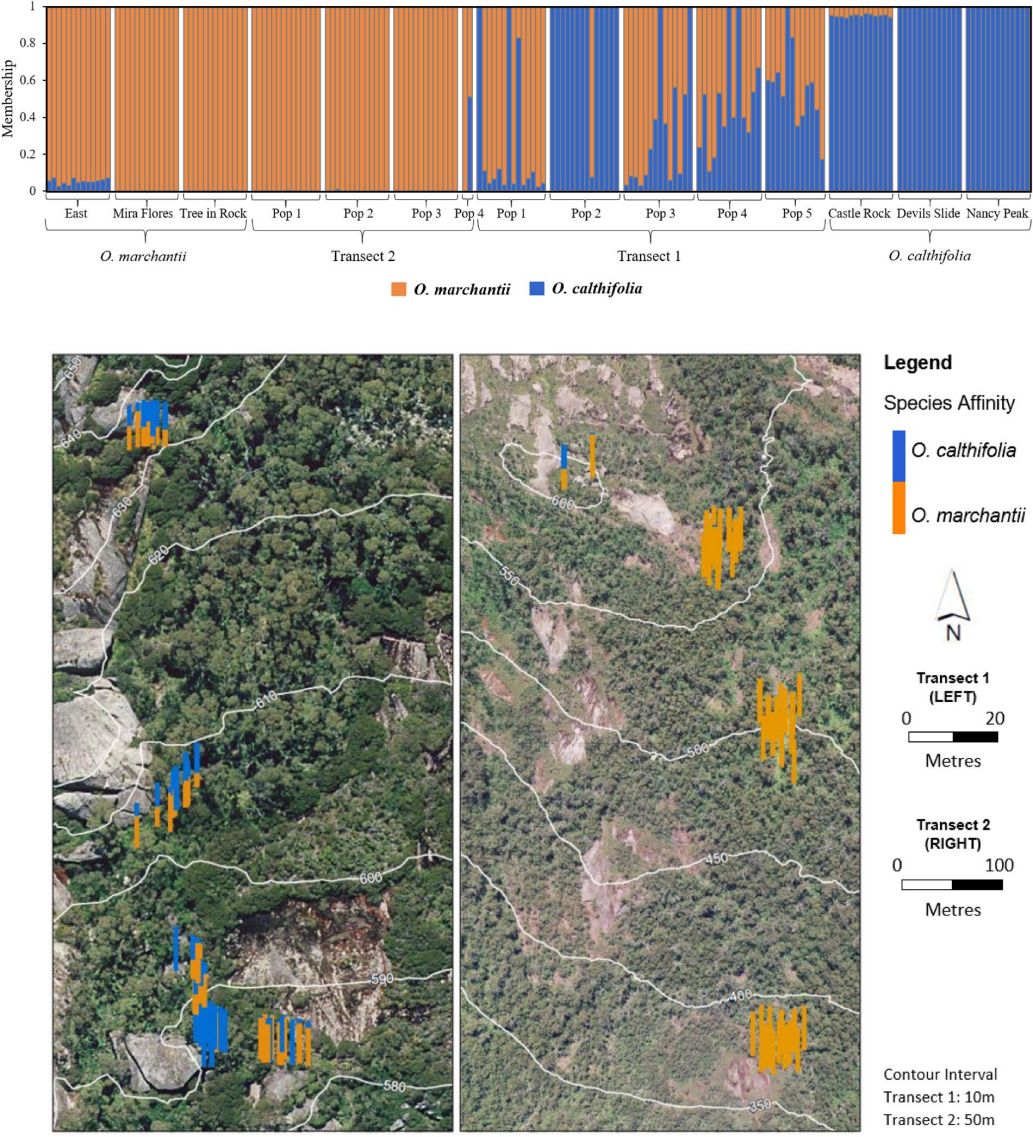
Population genetic structure and its spatial variation for two species of *Ornduffia* (*O. calthifolia* and *O. marchantii*) and their hybrids in the Porongurup Range, south-western Australia. (**A**) Population genetic structure with genetic assignment of individuals to either *O. calthifolia* or to *O. marchantii* designated from Bayesian techniques implemented in STRUCTURE. Blue is likelihood of assignment to *O. calthifolia*, orange is assignment to *O. marchantii*. Populations are *O. marchantii* East (OME), Mira Flores (OMM) and Tree in Rock (OMT), *O. calthifolia* Castle Rock (OCC), Devil’s Slide (OCD) and Nancy Peak (OCN), Transect 1 with populations 1–4 (T1–5) and Transect 2 with populations 1–4 (T1–5). (**B**) Disturbed and undisturbed transect sampling locations with admixture among samples, as calculated by STRUCTURE, overlayed on an aerial photograph. Contour interval = 10 m. Imagery was taken in 2011 by airplane flying 1700–2200 m above the ground using a Digital Mapping Camera from Z/I Imaging and acquiring RGB spectral bands covering 425–515, 515–550 and 600–650 nm at a spatial resolution of 0.2 m. ArcGIS v 10.5 was used to create the map.

The ΔK method identified K = 2 to be optimal. Variation between replicate runs on STRUCTURE did not result in any changes to species assignment when K = 2. Admixture modelling in STRUCTURE detected hybridisation between *O. calthifolia* and *O. marchantii* (Fig. 5) and individuals identified as hybrids based on morphology. Hybridisation was more frequent on Transect 1 with 31 hybrid individuals identified, with the remaining individuals identified as pure individuals of both parent species, except for a single hybrid found on Transect 2. All other individuals on Transect 2 were assigned to *O. marchantii*. Ninety-nine per cent of private alleles (alleles found only in one species) were shared with hybrids.

All samples from identified *O. calthifolia* populations were above the threshold of 0.9 and classified as pure. Most of the samples from populations of *O. marchantii* were also identified as pure, except for three samples of *O. marchantii* from the ‘OM East’ population, which were below the 0.9 threshold. We sampled this population at two slightly disparate locations (Fig. 2), with all seven samples from one location assigned a membership of approximately 0.9 to *O. marchantii*. Morphometric analysis suggested these samples were consistent as being from *O. marchantii* and these samples were considered as pure *O. marchantii* for SDMs.

Samples from Transect 1 showed a range of genetic affinities with some individuals assigned to each of the two parent species and the remainder showing a range of admixture. Samples assigned to either of the two parent species were located primarily at lower elevation, with assignment to *O. calthifolia* tending to increase with elevation along Transect 1 (Fig. 5). Further, each population along Transect 1 consisted of at least one pure *O. calthifolia*. The T1, 2 population was comprised of pure *O. calthifolia*, and occurred near a large cliff, which is habitat suitable only for *O. calthifolia* (as identified by SDM).

Results from NEW-HYBRIDS closely matched those from STRUCTURE. F_1_ and F_2_ Hybrids were more prevalent at higher altitudes along Transect 1. Across the five populations of increasing elevation, the number of F_1_ and F_2_ hybrids were 0, 0, 2, 7 and 7. Perfect classification of simulated individuals from HYBRIDLAB was achieved in both STRUCTURE and NEW-HYBRIDS with 100% of the simulated individuals correctly classified.

### Spatial patterns of hybridisation

**Figure 6:**
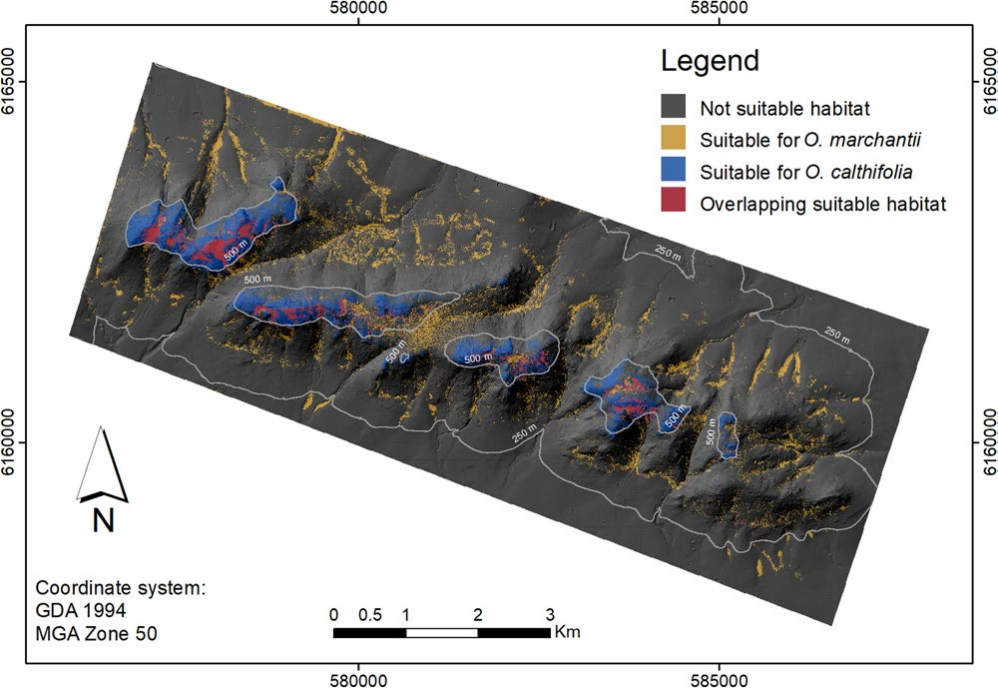
Species distribution model (SDM) identifying suitable habitat for two species of *Ornduffia* (*O. calthifolia* and *O. marchantii*) in the Porongurup Range, south-western Australia. Potential for natural hybridisation exists in the area of overlap (magenta). Threshold of suitability threshold at value producing highest overall accuracy. Area of overlap = 0.11 km^2^. Base imagery is from a Leica ALS 50-II scanner flown at 1700–2200 m, in 2011, and scanning approximately 1.5–2 km wide swaths. Triangulation was used to interpolate to a resolution of 2 m. ArcGIS v 10.5 was used to create the map.

Average area under the curve (AUC) statistics across 100 bootstrap runs based on a 20% subset for validation were 0.98 and 0.92 for *O. calthifolia* and *O. marchantii*, respectively. With elevation as the highest contributor (Table 2), distribution models of *O. calthifolia* suggest limitation to the summits or peaks, with more than 90% of the 275 ha of suitable area being above 500 m (Fig. 6).

**Table 2:** Summary statistics (mean, standard deviation and variable importance in the species distribution model; SDM) of each explanatory variable constructed using the same dataset as used in the SDMs (see text). Only confirmed hybrids from this study were used.

	DEM	SLO	ASP	CUR	SR	TWI	ROU	VDC	SWI	TPI	D2E	CH
*O. calthifolia* (n = 266)	596.4 (45.9)	29.7 (13.8)	185.3 (77.5)	−9. (42.6)	1119649.3 (249687.5)	3.9 (1.5)	1.1 (1.3)	0.4 (0.8)	1.0 (1.0)	10.8 (10.7)	4.7 (6.2)	1.9 (2.5)
	73.7%	0.3%	0.3%	0.0%	1.5%	0.1%	0.3%	0.0%	0.5%	1.2%	14.4%	7.8%
*O. marchantii* (n = 151)	416.8 (134.9)	13.2 (8.7)	141.5 (71.4)	−2.8 (7.7)	1274897.9 (152906.1)	5.6 (2.0)	0.37 (0.3)	0.1 (0.2)	2.6 (0.9)	−2.5 (3.1)	53.9 (95.7)	11.1 (9.6)
	29.3%	12.7%	4.8%	0.3%	1.9%	0.5%	1.0%	0.7%.	2.9%	21.7%	14.0%	10.2%
Hybrids (n = 32)	608.6 (17.8)	21.9 (10.6)	152.8 (26.7)	−16.6 (24.6)	1100719 (161067)	5.3 (2.4)	0.65 (0.38)	0.01 (0.03)	1.3 (0.7)	−3.4 (4.7)	3.8 (4.4)	2.3 (1.6)

Explanatory variables are elevation (DEM), slope (SLO), aspect (ASP), curvature (CUR), annual solar radiation (SR), topographic wetness index (TWI), roughness (ROU), vertical distance to channel network (VDC), SAGA wetness index (SWI), topographic position index (TPI), distance to edges (D2E) and canopy height (CH).

Analysis of explanatory variables showed elevation had less impact on the distribution of *O. marchantii*, and suitable areas of habitat can be found across the extent of the range. The extent of suitable habitat for *O. marchantii* (322 ha) was greater and far more widespread and fragmented than that of *O. calthifolia* (Fig. 6). The area of estimated suitable habitat intersecting both species was limited to approximately 59 ha, near mountain summits due to the elevation dependency of *O. calthifolia*. All hybrid individuals existed or were contained within the area apparently suitable for both species.

## Discussion

This study provides an exemplar of combining genetic data and spatial modelling approaches to enable more nuanced understanding of hybridisation among taxa of conservation significance. Genetic analysis clearly demonstrated presence of individuals from both species and individuals with a range of admixture. SDM identified the differences in suitable habitat, as well as an area of overlap suitable for both parents that is concurrent with the location of hybrids detected in genetic analyses.

### Genetic and morphological evidence of hybridisation

Hybridisation between *O. calthifolia* and *O. marchantii* was confirmed by combining morphological and genotyping approaches, supporting previous claims of putative hybrids^25,28^. Morphometric analysis identified phenotypic intermediates between the two species. However, high overlap with parental morphology made it difficult to definitively identify hybrid individuals and pure individuals along Transect 1. Nevertheless, admixture modelling in STRUCTURE based on genotyping conclusively identified 32 hybrid individuals, along with pure individuals of the two species.

An area of 59 ha suitable for both species was derived by intersecting SDMs of the parents to highlight the potential for co-occurrence and natural hybridization. All the Transect 1 sites and Transect 2, 3–4 were located within this overlap area. Therefore, the greater rate of hybridization may be due to greater overlap of the parent species in transect 1. However, given transect 1 is adjacent to a walking track it is possible that disturbance is opening new corridors for species contact and altering plant phenology, but this theory needs further exploration^60,61^. This is consistent with studies that show disturbance can affect the phenology of plants to induce the hybridisation (e.g.^61^), as opposed to merely bringing the taxa into contact (e.g.^62^). Plastic responses to habitat change (e.g. flowering time), are known to vary under conditions of anthropogenic disturbance^63,64^. Ornduff^20^ noted that there is some natural overlap in flowering and pollination times and was able to produce hybrids under laboratory conditions.

Whilst hybrid individuals were largely restricted to Transect 1, which is located within relatively disturbed habitat, our sampling was not designed to determine whether disturbance drives hybridization. However, given that the study site is a prominent tourist destination, future studies should investigate the role of disturbance in hybridization. Such studies could be complicated by a marked drop in the abundance of *O. calthifolia* since a 2013 study^22^. The rarity of this species therefore makes rigid sampling along elevational transect difficult. Limitations in study design mean we cannot rule out subtle differences in microhabitat between the two transects or that Transect 1 is located on a historic, geographically stable hybrid zone.

### Spatially variable introgression

We found evidence of introgression and backcrossing, as hybrids exhibited a range of affinity to each species. Without introgression, all hybrids would be expected to be F_1_ hybrids, and would display approximately 50% affinity to each parent^65^. Affinity to *O. calthifolia* increased with elevation in Transect 1 from the lowest population (median admixture = 0.07 *O. calthifolia*, 0.93 *O. marchantii)*, to the highest (median admixture = 0.59 *O. calthifolia*, 0.41 *O. marchantii*). This change in admixture is consistent with the bounded hybrid superiority model^66^, whereby a zone of hybridisation occurs between two geographically separated species, creating a gradient of admixture. In this model, hybrids possess superior adaptability to the intermediate conditions, but parent species dominate in their niche habitats^66^.

Numerous ‘pure’ individuals persisted within the hybrid zone of Transect 1, particularly in population 2, which contained almost exclusively ‘pure’ *O. calthifolia* individuals. Persistence of *O. calthifolia* amongst hybrids in this population is noteworthy considering the proximity of hybrids. The site was adjacent to a sheer rock face (Fig. 5), a key habitat property of *O. calthifolia*^22^. However, such formations were also present at populations comprised of hybrid individuals. SDM analysis showed that habitat in this area was no more suitable than other locations along the transect suggesting the influence of other factors such as soil depth.

### Implications of hybridisation

There are several potential positive and negative implications of hybridisation to the parent populations, but all are uncertain. For example, hybridisation may provide *O. calthifolia* with new genetic variation, alleviating inbreeding or facilitating response to changing abiotic conditions^67–69^. Alternatively, hybridisation may lead to genetic swamping of *O. calthifolia*^2,3^. In this study, hybrids occurred at elevations spatially associated with the restricted *O. calthifolia*, and as we found evidence for introgression, this species could potentially be at risk of genetic swamping^2,70^. Early generation hybrids identified at higher altitudes suggest invasion of *O. marchantii* into the higher elevation habitat of *O. calthifolia*. The expansion of *O. marchantii* into these higher elevations fits the climate induced altitudinal migration of species, which can result in swamping^1,3,71^. However, putative hybridisation has been suspected at this location for decades^18,20^, yet little progression in extent has been recorded. Laboratory tests found pollen viability of hybrids was approximately 50%, compared to >90% for pure specimens^20^. Therefore, it is possible that reduced fertility of hybrid individuals could slow or negate any swamping process. Moreover, the presence of pure *O. calthifolia* at population 2 on Transect 2 population demonstrates its ability to persist in niche habitat amongst hybrid populations.

The combination of genetic data and spatial modelling approaches enabled a better understanding of hybridisation among taxa of conservation significance. Although the hybrids were at the margin of suitable habitat for *O. marchantii*, their preferred habitat was closer to that of the more narrowly distributed *O. calthifolia*. The extent to which hybrid spread and competition for habitat presents as a threat to *O. calthifolia* is unknown, as is the role of disturbance in hybridisation. However, given the restricted distribution *of O. calthifolia* (endemic to high elevations in the Porongorups) and the threats posed by global warming and disturbance through tourism, understanding the dynamics of hybridisation should be a priority in monitoring and conservation management.

## Data Availability

All genotype data will be available from the Dryad data repository.
